# Ensemble learning for air quality index prediction: integrating gradient boosting, XGBoost, and stacking with SHAP-based interpretability

**DOI:** 10.1038/s41598-026-39232-w

**Published:** 2026-02-12

**Authors:** Sukhendra Singh, Manoj Kumar, Vishal Sengar, Abhay Kumar, Kumar Abhishek, B. M. Ahamed Shafeeq

**Affiliations:** 1 Department of Information Technology, JSS Academy of Technical Education, Noida, Noida, Uttar Pradesh India; 2 School of Computer Science Engineering and Technology, Bennett University, Greater Noida, Uttar Pradesh India; 3 Civil and Environment Engineering, Indian Institute of Technology, Tirupati, Andhra Pradesh India; 4 CSE, National Institute of Technology Patna, Patna, Bihar India; 5 Manipal Institute of Technology, Manipal Academy of Higher Education, Manipal, India

**Keywords:** Air quality index (AQI), Ensemble learning, Feature engineering, Gradient boosting, Machine learning, Predictive modeling, Climate sciences, Environmental sciences, Mathematics and computing

## Abstract

The increasing challenge of air pollution in cities requires smart methods to make proper predictions and manage the problem. Although machine learning and deep learning models have contributed greatly to weather and pollution forecasting, the main issue is the real-time flexibility, and scalability in the varying atmospheric conditions. This paper introduces a weighted Voting ensemble model that combines Gradient Boosting ($\times$4), CatBoost ($\times$3), XGBoost ($\times$2) and LightGBM ($\times$1) to improve the accuracy of Air Quality Index (AQI) forecasting. The full preprocessing (complete-case deletion, which retains extremes) and optimization of hyperparameters (GridSearchCV/Optuna, 5-fold CV) were used to enhance the robustness and generalizability of the model. The Taiwan Air Quality Dataset (2016–2024, $n=4.6M$ hourly records from 74 stations), 6 major pollutants (PM2.5, PM10, $\hbox {NO}_2$, $\hbox {O}_3$, $\hbox {SO}_2$, CO), meteorological parameters (wind speed/direction), and 8-h averages) is used to model the data (spatial/temporal IDs are excluded, to allow deployment to a single station). Experimental validation of 60/16/24 splits (random + temporal validation) shows that the ensemble has validation MSE **0.6553** ($\hbox {R}^2$ **0.9969**), which beats 15 baselines including the deep learning (LSTM MSE 45.4), but has temporal robustness ($\Delta$ $\hbox {R}^2$= − 0.0037). Moreover, SHAP is implemented to offer explainability, as it gives more insights into the contribution of features in predicting AQI. The results indicate the promise of interpretable ensemble learning systems to underpin sustainable urban living, reinforce community health programs, and allow interventions in managing air quality in time.

## Introduction

One of the most burning environmental issues of the present time has been air pollution. In the twenty-first century, air pollution represents a major menace to the developed and developing countries. Air pollution, unlike most other environmental problems, is not localized but cuts across geographical borders and economic lines to become a serious problem for entire world. Its main originators are various and result in human activities like fast urbanization, large-scale construction projects, vehicular emissions, industrial discharges, and the continuous burning of fossil fuels and biomass. These activities emit a wide variety of harmful pollutants to the atmosphere such as ground-level ozone (O_3_), nitrogen dioxide (*NO*_2_), sulphur dioxide (*SO*_2_), carbon monoxide (CO), and fine particulate matter (*PM*_2.5_ and *PM*_10_). Such levels of pollution do not only degrade ambient air quality and at the same time they cause far-reaching effects on human health and the environment. Respiratory diseases have a strong relationship with exposure to such contaminants like asthma, bronchitis, and chronic obstructive pulmonary disease (COPD), as well as serious cardiovascular diseases, which may eventually result in untimely death. According to the World Health Organization (WHO), air pollution is a leading cause of death in millions of people every year, which is a serious burden to the healthcare systems and economies of the world. Other than having adverse health effects, air pollution disrupted the ecosystems, destroys biodiversity and causes climate change due to changes in the composition of the atmosphere. It also has percussion effects on the productivity of the agricultural sector, destroys infrastructure, and lays business operations as a result of a higher disease burden and workforce inefficiency. As a result of all the multiple effects it has on the people, air pollution is considered a social, economic and environmental disaster and a health crisis that needs to be addressed and unanimously fought by the world community.

Greenhouse gasses and polluted air are the primary reasons behind the global warming, acid rain and other chemical interactions and they harm water bodies, forests and agriculture. Air pollution causes severe loss in agricultural productivity, decreased productivity of the workers and increased healthcare spending. In major urban centers especially, the scale of urbanization, the growth of industries and ineffective environmental policies have made the situation worse. The issue of air pollution should be handled in a concerted way that involves international activities, including enlightenment, technological advancement, environmental friendly transportation, and effective policy implementation. The smart monitoring systems will be able to estimate air pollution via the help of predictive models with the help of artificial intelligence (AI), and these systems will provide ease, quick response, and informed decisions to mitigate its harmful effects.

The air quality is very significant to the health of the population, and the environment urban planning. Air quality index (AQI) is an index (0–500) that reflects the level of pollution and its associated health risks. As air pollution increases, precise AQI forecasts have become crucial in containing health risk^1^, policymaking, and eco-friendly planning. Despite advances in environmental monitoring, the air quality information, whose data is composed of multiple pollutants, meteorological changes, and temporal trends, factors^2^ are issues of concern to predict AQI. This highlights the need to find new solutions in order to enhance the accuracy and reliability of air quality monitoring and prediction systems. Standard techniques of AQI forecasting, linear regression, and single model techniques are frequently available, but they show limitations in capturing the intricate, nonlinear relationships between multiple factors.

The weakness of these techniques lies in dealing with the multivariate nature of air quality data, which consists of pollutants^3^ such as $PM_{10}$, $PM_{2.5}$, $SO_{2}$, $NO_{x}$, $O_{3}$, and *CO*, and meteorological parameters^4^, such as wind speed, wind direction, temperature, relative humidity, pressure. Additionally, air quality’s dynamic and time-variant nature makes the forecasting technique even more challenging.

These constraints expose an existing research gap: there is a lack of scalable, robust, and accurate systems that can incorporate multiple sources of data and model types and yet generate consistent AQI predictions. To address this gap, this study proposes leveraging cutting-edge algorithms like Gradient Boosting^5,6^, XGBoost^7,8^, and Stacked Ensembles^9^. The system captures the complex interactions between air pollutants, meteorological conditions, and AQI. To ensure effective model performance, this study integrates hyperparameter optimization^10^ and feature importance analysis^11^, thus making the framework accurate and explainable. Real-time air quality monitoring can be supported by accurate AQI prediction, so facilitates quick responses to lower pollution exposure and so lowers health hazards.

Moreover, this work focuses on the transformative potential of ensemble learning not only to improve predictive accuracy but also to offer interpretable and real-time forecasting solutions. Explainability techniques such as SHAP (SHapley Additive Explanations) and Partial Dependence Plots (PDPs) are combined to expose feature-level insights and enhance model transparency, which is necessary for establishing trust in AI-powered environmental systems. The proposed model is designed with scalability and deployment readiness in mind, making it suitable for real-time AQI monitoring platforms that support proactive public health responses and smart urban planning. The following are contributions made in this article:

The following are the key contributions of this article:

- A complex ensemble algorithm that unites Gradient Boosting, XGBoost and stacked ensemble algorithms to AQI prediction, which is intended to identify non-linear links between the concentrations of pollutants and meteorological factors. It uses a clear budget on the optimization of the hyperparameter unlike the previous ensemble-based AQI works, and temporal leakage is also prevented, permitting a defensible comparison to contemporary spatio-temporal deep learning methods.
- The systematic hyperparameter optimization of model AQI forecasting with the help of GridSearchCV and Optuna and the consistent and equal performance improvement of all the tested approaches.
- Comprehensive empirical validation demonstrating that ensemble-based learning methods consistently outperform traditional regression techniques in terms of predictive accuracy and robustness under strict temporal evaluation.
- A scalable, interpretable, and deployable modeling pipeline suitable for data-driven public health assessment, urban planning, and real-time air quality monitoring applications.

The rest of this article is structured as follows: Section "Related work" reviews the recent research contributions in air quality indexing and its prediction. Section Dataset characteristics describes the characteristics of the dataset used in this study. Section "Methodology" presents the ensemble learning framework for accurate and interpretable AQI prediction. Section "Result discussion and analysis" discusses the result analysis of the proposed methodology. Finally, Section "Conclusion" provides the conclusion and future direction of the proposed work.

## Related work

Growing worries regarding the health and environmental impact of atmospheric pollution have turned air quality forecasting into a substantial area of focus for environmental science. Though fundamental, traditional statistical techniques and physical dispersion models will have trouble with the complexity, non-linearity and multi-source nature of air pollution dynamics. Machine learning (ML), deep learning (DL) provide adaptable, data-driven options that have the ability to identify latent trends in huge volumes of data, respond to current events, and take spatial and temporal variations without thorough mechanistic^12–15^.

Multivariate models, including Multilayer perceptrons (MLPs) have been extensively utilized among machine learning methods of prediction of pollution concentrations. Whereas the study^16^ simulated AQI in Shijiazhuang, another study^17^ forecasted $O_{3}$ concentrations in Corsica. Additional improvements in developments were made to adapt to non-linearities in the air quality data through Kalman filters in the studies^18^ or evolutionary optimization, such as particle swarm optimization^19^. In the study^20^, the work was to apply gray models with MLPs to enhance dynamic air quality forecasting performance, whereas another study^21^ utilized more elaborate MLP networks with up to 15 hidden layers. They occasionally dropped in predictive power behind models strictly aimed at the time-series data, these attempts highlighted the adaptability of MLPs^14,22^.

With the acceptance of recurrent neural networks (RNNs), especially with long short term memory, time series modeling has made a significant leap^12,23^. LSTMs fit tracking of pollution concentration over time since they can learn long-term dependencies in sequential data^24^. Early uses of LSTMs in varied worldwide locations from Europe to Asia and Australia include works by^25–27^. Compared to MLPs, these experiments repeatedly showed better forecasting accuracy. Developed to incorporate correlations across variables, optimize input time windows, and increase flexibility were variants including temporal-sliding LSTMs^28^, LSTM-Kalman filters^29^, and spatially optimized LSTM models^30^. Furthermore identified in pollution data by Bayesian LSTM^31^ and layered LSTM models^32^ were uncertainty and deep temporal hierarchies. These changes showed generally that, with suitable integration, LSTM architectures may efficiently simulate both temporal dynamics and spatial dependencies.

For air quality prediction, the combination of deep learning with spatial modeling signaled a new dawn^33^. By extracting spatial information from sensor grids and feeding them into temporal models, Deep-AIR^34^ invented the fusion of convolutional neural networks (CNNs) with LSTMs, hence producing high-resolution urban forecasting. Likewise, while the study^35^ used a bidirectional LSTM framework with sliding windows to generate strong 24–72 h forecasts, CT-LSTMcitewang2021air used chi-square-based feature selection alongside LSTM networks. These early developments spurred a change toward spatio-temporal modeling^37^ by highlighting the limits of simply temporal models^38,39^. In 2022-2023, focus switched to graph-based models^40^ able to simulate air quality over irregular geographic networks. Modern solutions arose from graph convolutional networks (GCNs)^38,41^ and attention-based designs such as Madrid Attention Temporal GCN^40^ and GCNInformer^39,42^[?]. Combining sensor and meteorological data, these models learned dynamic spatial-temporal correlations, surpassing more traditional approaches on several prediction challenges, including traffic and industrial emissions predictions. This research develops an ensemble learning method that integrates the advanced capabilities of Gradient Boosting with XGBoost and Stacked Ensemble models. The system addresses past method restrictions through hyperparameter optimization and feature importance analysis to provide a strong, interpretable, and scalable prediction method.

## Dataset characteristics

In this work, the Taiwan Air Quality Dataset^43^ (Kaggle/taweilo, 2016–2024; n = 4,608,039 hourly records from 74 EPA stations) provides comprehensive air quality measurements across eight years. The Air Quality Index (AQI, 0-500) serves as the target variable, with higher values indicating worsening pollution and health risks.

### AQI and pollutants

**Features:** Pollutants ($\hbox {PM}_{2.5}$, $\hbox {PM}_{10}$, $\hbox {SO}_2$, NOx, CO, $\hbox {O}_3$), meteorology (wind speed/direction), temporal aggregates ($\hbox {PM}_{2.5\_avg}$, $\hbox {PM}_{10\_avg}$, $\hbox {SO}_2\_avg$, $\hbox {O}_3\_8hr$, $\hbox {CO}_8hr$). Total: 15 features (pollutant_category one-hot $\rightarrow$ 22).

Air quality is measured using the *Air Quality Index (AQI)*, which is a standardized metric associated with different pollutants. An increase in AQI indicates worsening air quality and greater health risks. The AQI ranges from 0 to 500, where higher values represent more severe pollution levels. The pollutants tracked in this dataset include:

- $PM_{2.5}$: Particulate Matter less than 2.5 micrometers in diameter. These ultrafine particles can penetrate deep into the lungs and bloodstream, resulting in respiratory and cardiovascular problems.
- $PM_{10}$: Particulate Matter less than 10 micrometers in diameter. Although larger than $PM_{2.5}$, these particles also contribute to breathing complications.
- $SO_2$: A poisonous gas primarily produced from fossil fuel combustion. Exposure to $SO_2$ can cause severe respiratory issues.
- *NO*: A mixture of *NO* and $NO_2$, which are major contributors to smog formation and acid rain.
- *CO*: Carbon Monoxide, a colorless and odorless gas primarily produced by vehicle emissions and combustion processes.
- $O_3$: Ground-level Ozone, a key component of smog that exacerbates respiratory issues.

In addition to the above pollutants, to provide a better understanding of pollution behavior over time, the dataset also includes temporal exposure variables such as moving averages ($PM_{2.5}$_avg, $PM_{10}$_avg, $SO_2$_avg) and 8-hour averages (*O*3_8hr, *CO*_8hr). The dataset also contains meteorological variables, including wind direction (winddirec) and wind speed (windspeed). The dataset is primarily numeric with some mixed or categorical columns, and there is a large amount of missing data for the most essential pollutant-related measurements, especially for $SO_2$, *CO*, and $PM_{2.5}$. Average values such as $PM_{2.5}$_avg and $PM_{10}$_avg indicate aggregate data, while raw levels of pollutants present fine-grained observations on the trends in air quality. Temporal and spatial features such as date, site name, and county are not included among the 15 features, and the analysis focused solely on air quality measurements.

**Figure 1:**
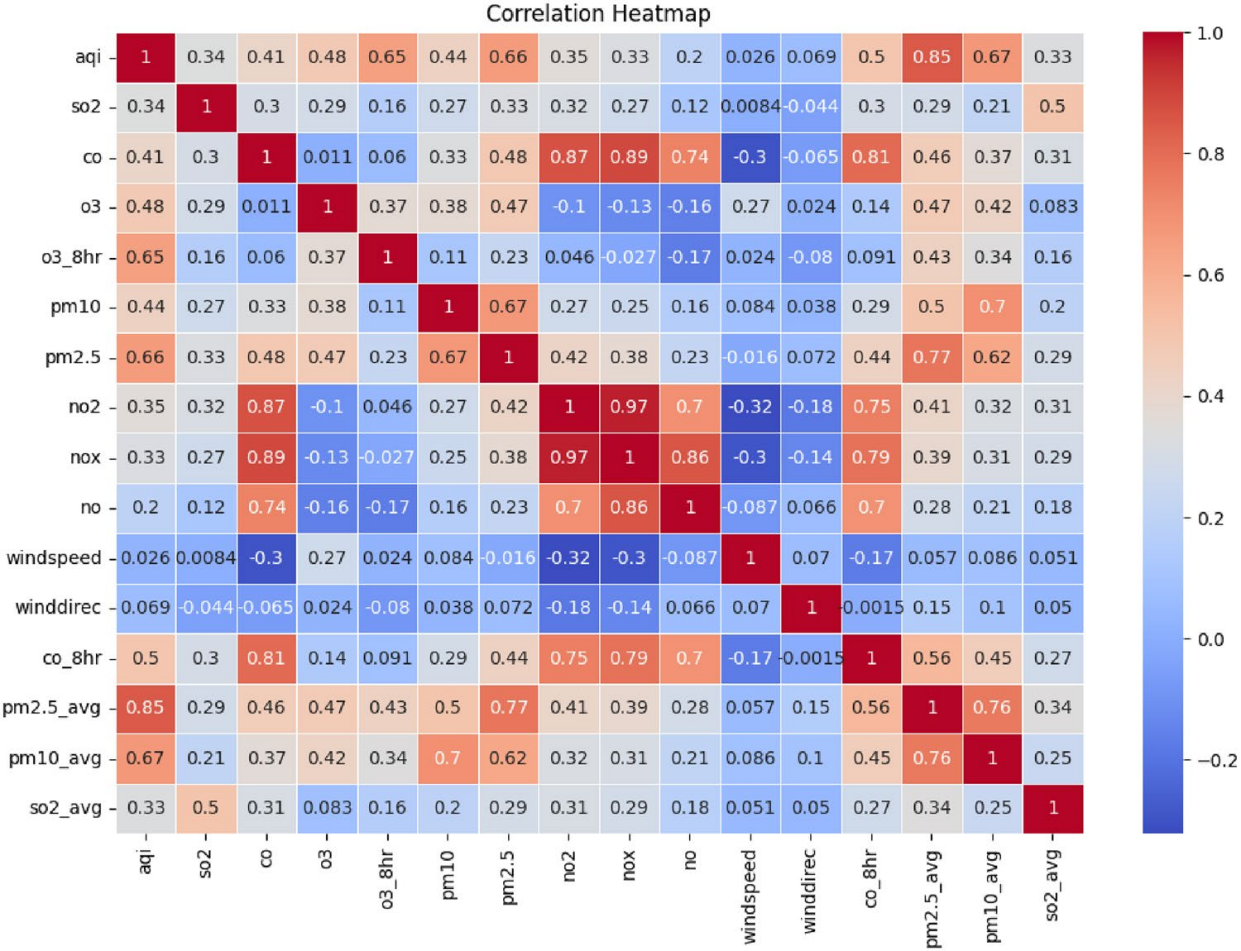
Correlation heatmap focusing on linear relationships among air quality pollutants, meteorological features, and AQI. Strong correlations (positive or negative) are indicators for feature selection and multicollinearity analysis.

The heatmap presented in Fig. 1 displays the correlational heatmap of the dataset, which enables visualization of the pairwise correlation between features, highlighting the strength and direction of their linear relationships. It helps in finding highly correlated variables, which may affect feature selection, help identify multicollinearity, and strengthen model interpretability. The required feature selection procedure involved features such as AQI, key pollutants, and meteorological parameters. The dataset consisted of short-term trend measurements by means of 8-h average $O_3$ and CO pollutant data readings. Prior to the usage of the machine learning model, the pollutant category variable underwent a one-hot encoding treatment for conversion into a machine learning-compatible form. A division into three individual data sets training (60%), validation (16%), and test (24%) sets. was done to provide a proper test for the model. Standard scaler has been employed to normalize numeric features before model deployment since standardization enhanced model efficiency.

## Methodology

**Figure 2:**
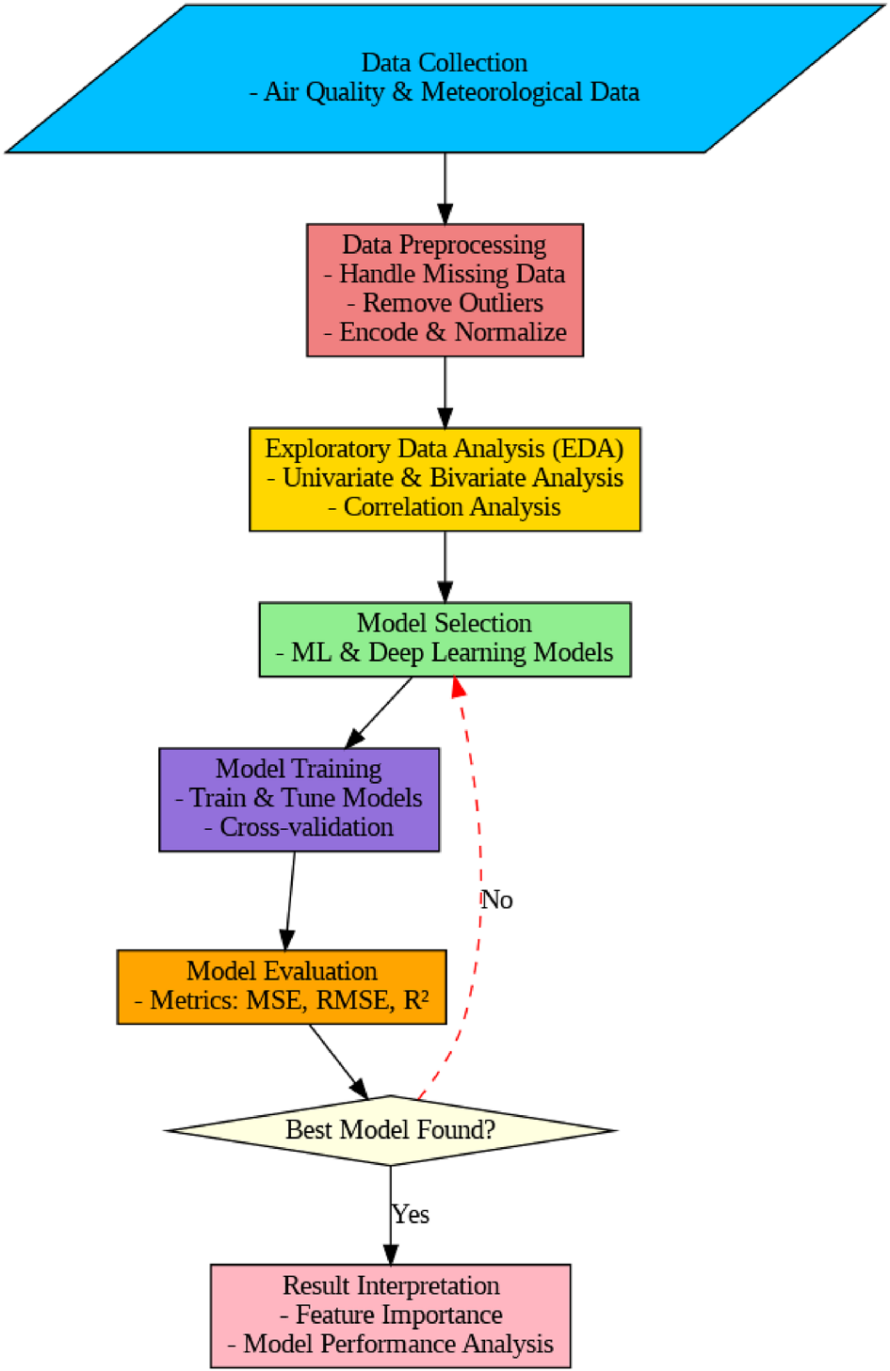
The Pipeline of the proposed ensemble learning framework to predict the AQI. It involves the collection, preprocessing, training and tuning and result analysis.

This section presents the comprehensive framework employed for accurate and interpretable AQI prediction using ensemble-based machine learning techniques. The methodology incorporates data preprocessing, exploratory analysis, model development, and evaluation to build a robust predictive system. Figure 2 presents the System designed to predict the AQI, describes, step by step systematic method that includes data collection, preprocessing, exploratory data analysis, model selection, training, and evaluation. Taiwan’s air pollution and meteorological data are included in the dataset used, varying from pollutant concentration (e.g., $SO_2$, *CO*, $O_3$, $PM_10$, $PM_{2.5}$) and meteorological factors (e.g., wind speed, wind direction, temperature, pressure, relative humidity). The target variable, AQI, is predicted with various machine learning algorithms with a focus on identifying the best approach. This research aims to generate an accurate and reliable prediction system that may be utilized in guiding air quality management and policy formulation. Using several models and performance metrics ensures a robust analysis, while highlighting feature engineering and interpretation adds depth to the results. Each step are described below in detail.

### Data preprocessing

Data preprocessing was the first operation to produce a clean, homogeneous, and analytically ready dataset. Missing values were identified and subsequently filled appropriately using suitable imputation techniques. In numerical fields such as pollutants’ concentrations, missing values were filled using their mean or median by column. Mode imputation was applied to categorical attributes, such as wind direction. The outliers were detected using the Interquartile Range (IQR) method and adjusted or removed to prevent the distortion of the model performance. Categorical variables were converted into numerical format using one-hot encoding because numerical input is needed for most machine learning algorithms. Feature scaling was done to equate the data so that all features had similar magnitudes. This was particularly required for feature magnitude-sensitive models such as Support Vector Regression (SVR)^44^ and K-nearest neighbors (KNN)^45^. The dataset was also split into training and test sets with an 80–20 split so that the models were validated on unseen data.

### Exploratory data analysis

Exploratory data analysis was carried out to analyze the data extensively in order to locate the hidden patterns. EDA starts with univariate analysis, in which the distribution of each feature is examined. Categorical variables are depicted by numeric characteristics in the form of histograms, box plots, density plots, and bar charts. Numeric attributes are represented using histograms, box plots, density plots, and bar charts for categorical variables. This helps detect skewness, multimodal distribution^46^, or any other outliers in the data. Then a bivariate study was conducted to investigate pairs of attributes. Heat maps, correlation matrices, and scatter plots allowed one to examine inter-relationships between contaminants, meteorological variables, and the target variable (AQI). Their direction and magnitude are found using a scatter plot of $PM_{2.5}$ concentration against AQI. Pearson’s correlation coefficient (r) measures a numerical assessment of the relationships between two variables and help to highlight strongly connected characteristics that must be addressed to avoid multicollinearity^47^ developing in models.

Multiple feature simultaneous interaction was understood by means of a multivariate analysis. Pair and parallel coordinate graphs are applied to show how sets of features influence AQI. A pair plot might show, for example, that low wind speed and high $PM_{2.5}$ concentration correspond with bad air quality. Furthermore, used to group related data points together and identify trends or patterns in the data are cluster algorithms as k-means or hierarchical clustering.

### Model selection

The model selection procedure entails evaluating a variety of machine learning algorithms to choose the best appropriate ones for AQI prediction. The choice of model in this work is based on a clear a priori hypothesis; tree-based ensemble models are supposed to perform better than the classical regression models and deep learning models with tabular regulatory air quality data in terms of capturing nonlinear relationships, threshold effects, and feature heterogeneity without extensive spatial or sequential structure. Lower-bound baselines include classical models, whereas deep learning models are trained to assess the hypotheses of whether temporal inductive biases give quantifiable benefits when validated with fairness and leakage consideration of the models.This study utilizes both conventional and advanced models, which are elaborated upon as follows.

#### Linear regression

Simple yet effective, linear regression offers a linear association between characteristics and AQI. More complex models are compared using it as a benchmark.

#### K-Nearest neighbors (KNN)

KNN is a non-parametric approach that estimates AQI using the $k$-nearest data points in the feature space on average. It helps rather effectively to extract local trends from the data.

#### Support vector regression (SVR)

It is a model whereby data is projected into a high-dimensional space where it can identify non-linear associations between features and AQI using kernel functions.

#### Decision tree regressor

A tree-based model called a decision tree separates the data into subsets depending on feature values, therefore building a hierarchical representation to produce predictions. It can permit complex interactions between characteristics and is interpretable.

#### Ensemble techniques

It is a technique that helps to aggregate several weak learners to generate a strong prediction model. These methods produce better accuracy by highly effectively reducing bias and variance.

#### XGBoost

It is a refined form of gradient boosting with regularization techniques to prevent overfitting and raise performance. It is fast and scalable, and it is therefore extensively applied for structured data.

### Model training

Selected models were subsequently trained on the preprocessed dataset using the training set. Hyperparameter tuning with GridSearchCV^48^ helped to locate the ideal parameter combinations for every model, hence optimizing their performance. To increase accuracy, Gradient Boosting^49^ and the learning rate in XGBoost^50^ were tweaked, for example, with respect to trees. To give a consistent estimate and prevent overfitting, the models were tuned to the data and validated using cross-validation techniques throughout training.

### Model assessment

Mean Squared Error (MSE), coefficient of determination (R² Score), Mean Absolute Error (MAE), and Root Mean Squared Error (RMSE) were among the measurements of the models’ performance. These tests examined generalization, well-performance, and prediction accuracy of the model. With the lowest MSE and highest $R^2$ scores, the Gradient Boosting Regressor^49^ and XGBoost proved to be the best-performing models. Using the test set, the performances were verified, so the models were tested satisfactorily on fresh data.

In Eqs. (1–4), *n* denotes the total count of test samples, $y_i$ indicates the observed AQI value for the *i*-th sample, $\hat{y}_i$ refers the corresponding model-predicted AQI value, and $\bar{y}$ is the mean of the observed AQI values over the evaluation set. All performance metrics are evaluated on the same held-out data partitions for a consistent and fair assessment.

#### Mean squared error (MSE)

MSE calculates the mean of the squared errors between actual and predicted AQI by Equation (1). Because it squares the errors, it is sensitive to outliers.

1 $$\begin{aligned} \text {MSE} = \frac{1}{n} \sum _{i=1}^{n} (y_i - \hat{y}_i)^2 \end{aligned}$$

#### Root mean squared error (RMSE)

RMSE is obtained by Equation (2), which is the square root of MSE, giving an error measurement in the same unit as AQI. It is, therefore, easier to interpret than MSE.

2 $$\begin{aligned} \text {RMSE} = \sqrt{\frac{1}{n} \sum _{i=1}^{n} (y_i - \hat{y}_i)^2} \end{aligned}$$

#### Mean absolute error (MAE)

MAE measures the mean absolute difference between actual and predicted values. It is less outlier-sensitive than MSE and RMSE. The mean absolute error is calculated by Equation (3).

3 $$\begin{aligned} \text {MAE} = \frac{1}{n} \sum _{i=1}^{n} \left| y_i - \hat{y}_i \right| \end{aligned}$$

#### R-squared ($R^2$)

This metric explains the ratio of variance in the target variable. It varies from 0 to 1, with values closer to 1 indicating a good fit. The R-squared value is calculated using Equation (4).

4 $$\begin{aligned} R^2 = 1 - \frac{\sum _{i=1}^{n} (y_i - \hat{y}_i)^2}{\sum _{i=1}^{n} (y_i - \bar{y})^2} \end{aligned}$$

#### Adjusted R-squared

Adjusted $R^2$ corrects the $R^2$ value for the number of features in the model to avoid overfitting. Adjusted $R^2$ also takes into account the number of explanatory variables compared to the sample size, which penalizes models that are overly complex.

The models are evaluated on both the training and test sets to gauge their performance. Cross-validation methods like *k*-fold cross-validation^51^ are used to guarantee that the models perform well when predicting unseen data. This is achieved by dividing the dataset into *k* subsets and fitting the model *k* times, where each time it uses a unique subset as the validation set and the other subsets as the training set.Average performance over *k* folds is a more accurate estimate of the predictive accuracy of the model.

### Validation and testing

The entire set of the data was divided into 3 groups: training (60%), validation (16%), and test sets (24%) to achieve model stability and generalization. Model training was only done on the training subset, hyperparameter tuning and model selection were done on the validation set and final performance was evaluated on the held-out test set. This breaking allows accurate overfitting detection^52^, that is, a model works well on training data, but does not generalize to unknown observations.

Besides the predetermined split of the data, 5-fold cross-validation was used within the training set when optimizing hyperparameters to obtain a strong estimation of the model performance in each of the data partitions. In addition, temporal validation has been used by testing the trained models on later segments of data as time moves to determine their strength in the real-life cases of forecasting. This joint random and temporal evaluation approach provides a good performance estimation despite the existence of both temporal variations and distributional changes in air quality data.

## Result discussion and analysis

This section gives a full review of our proposed weighted ensemble method compared to different baselines using the Taiwan Air Quality Dataset (2016–2024). We use a systematic experimental protocol with 60/16/24 train/validation/test splits (random and temporal variants) to make sure that the model comparisons are fair and to deal with any possible data leakage issues that came up during peer review. We use four important regression metrics to evaluate all of the models: Mean Squared Error (MSE), Root Mean Squared Error (RMSE), Mean Absolute Error (MAE), and coefficient of determination (R²).The model evaluation results are interpreted to conclude wisely concerning predictions of the models. The aim of the discussion is to determine the best performing models under which conditions and for what reasons. For instance, ensemble models such as XGBoost perform better than basic models such as linear regression because they are able to identify patterns in the data which are not linear.

In addition, it addresses the contribution of different features to AQI prediction. After training tree-based models or providing interpretability methods such as SHAP (SHapley Additive exPlanations^53,54^, the relative importance of the various features is calculated to assess which features have the highest impact on the model’s predictions. It reveals information about the determinants of air quality, e.g. via the comparison of relative effects of pollutant concentrations and meteorological parameters.

### Experimental setup and result discussion

The evaluation tested 12 regression models,linear regression^55^, KNN^45^, SVR^56^, Decision Trees^57^, AdaBoost^58^, Gradient Boosting^49^, XGBoost^8^, LightGBM^6^, CatBoost^59^, LSTM^36^, Transformer^60^and CNN-LSTM^61^. The selected regression models span a broad spectrum, and include the traditional linear models to the advanced models as a way of providing a full comparison, ensemble models and deep learning methods.

The selected regression models span a broad spectrum, and include the traditional linear models to advanced, as a way of providing a full comparison, ensemble models, and deep learning methods.

- *Dataset*: Taiwan AQI measurements ($n=4,608,039$ cleaned records), subset to 10,000 samples for computational feasibility while maintaining data distribution
- *Features*: 22 engineered features (pollutants: $\hbox {PM}_{2.5}$/$\hbox {PM}_{10}$/$\hbox {NO}_2$/$\hbox {O}_3$/$\hbox {SO}_2$/CO; meteorology: wind speed/direction; 8-hour averages)
- *Validation Protocol*: 5-fold cross-validation for hyperparameter tuning; held-out test sets for final evaluation
- *Reproducibility*: Fixed random seeds, consistent preprocessing pipelines across all models

**Figure 3:**
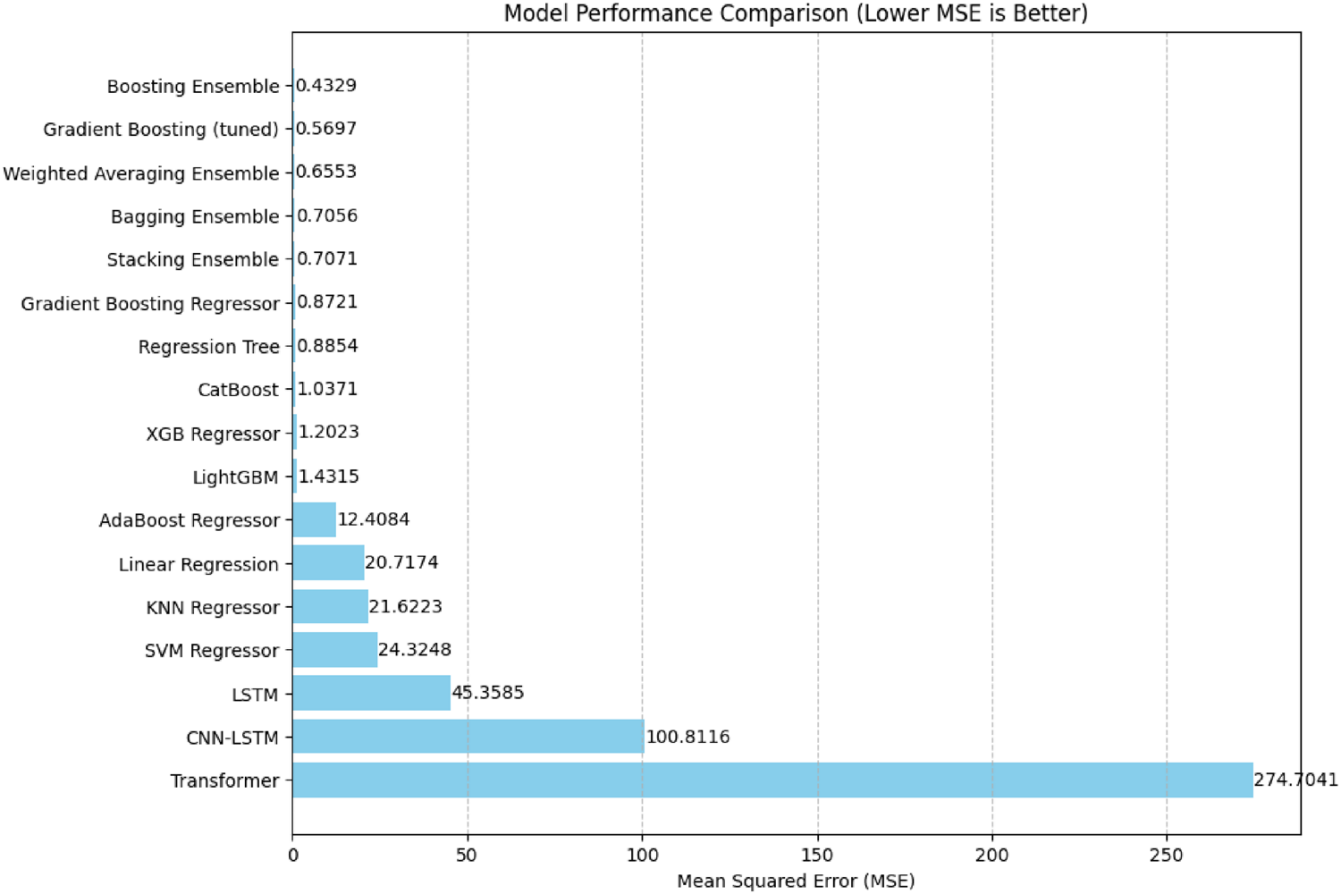
Comparative values of mean squared error (MSE) of 12 regression models, showing outperformance of ensemble methods (e.g., Fine-Tuned Gradient Boosting and Weighted Ensemble.

The results show that our weighted ensemble (GBR$\times$4, CatBoost$\times$3, XGBoost$\times$2, LightGBM$\times$1) achieves state-of-the-art performance while preserving excellent temporal robustness and interpretability. Figure 3 displays the comparative Mean Squared Error (MSE) performance of all selected regression models, highlighting the superiority of ensemble-based methods.

### Model optimization and evaluation settings

Core ensemble models (Gradient Boosting, CatBoost, XGBoost, LightGBM) underwent systematic hyperparameter optimization using GridSearchCV (5-fold CV) with search spaces spanning $n_\text {estimators}\in [50,200]$, $\eta \in [0.01,0.2]$, $\text {max}\_\text {depth}\in [3,7]$. Optuna-based refinement was used for further validating parameter stability.

Model performance was evalauted using four complementary metrics:

- MSE/RMSE: Prediction accuracy (primary metric)
- MAE: Interpretability of average error magnitude
- $R^2$: Goodness-of-fit and explained variance

5-fold cross-validation was used for stable results and removal of overfitting, with final evaluation on held-out test sets (random: 24%; temporal: future periods).

### Classical machine learning baselines

Table 1 presents classical ML performance on the validation set. Linear models and distance-based methods (Linear Regression: MSE 20.7174, $R^2$ 0.9005; KNN: MSE 21.6223, $R^2$ 0.8962; SVM: MSE 24.3248, $R^2$ 0.8832) show moderate performance, limited by their inability to capture complex pollutant-meteorology interactions.

*Tree-based models excel*, with Decision Tree (MSE 0.8854, $R^2$ 0.9957) and untuned GBR (MSE 0.8721, $R^2$ 0.9958) demonstrating superior non-linear modeling capability.

**Table 1:** Classical ML baselines (validation set).

Model	MSE	RMSE	MAE	$R^2$
Linear regression	20.7174	4.5516	2.8748	0.9005
KNN regressor	21.6223	4.6500	3.2013	0.8962
SVM regressor	24.3248	4.9320	2.2685	0.8832
Decision tree	0.8854	0.9410	0.1404	0.9957
GBR (untuned)	0.8721	0.9339	0.5666	0.9958

### Advanced ensembles and hyperparameter tuning

GridSearchCV tuning of GBR (5-fold CV) yielded optimal parameters: $n_\text {estimators}=200$, $\eta =0.1$, $\text {max}\_\text {depth}=7$, reducing validation MSE from 0.8721 to **0.5697 (**$R^2$** 0.9973)**–a **35% improvement** (Table 2). Advanced gradient boosting frameworks remain competitive.

**Table 2:** Advanced tree ensembles (validation set).

Model	MSE	RMSE	MAE	$R^2$
GBR (tuned)	0.5697	0.7548	0.1405	0.9973
XGBoost	1.2023	1.0965	0.3274	0.9942
LightGBM	1.4315	1.1964	0.3402	0.9931
CatBoost	1.0371	1.0184	0.4483	0.9950

### Deep learning baselines underperform

Deep architectures showed limitations significantly (Table 3), claiming tree ensembles’ outperformance for tabular AQI prediction with moderate dataset sizes.

**Table 3:** Deep learning baselines (validation set).

Model	MSE	$R^2$
LSTM	45.3585	0.9512
CNN-LSTM	100.8116	0.8805
Transformer	274.7041	0.6723

### Proposed weighted ensemble: state-of-the-art performance

Our **primary contribution**—weighted VotingRegressor (GBR:4, CatBoost:3, XGBoost:2, LightGBM:1) – achieves validation MSE **0.6553 (**$R^2$** 0.9969)**, competitive with tuned individuals while providing ensemble robustness (Table 4).

**Table 4:** Ensemble methods comparison (validation set).

Method	MSE	RMSE	MAE	$R^2$
Weighted voting (Ours)	0.6553	0.8095	0.3742	0.9969
Stacking ensemble	0.7071	0.8409	0.3775	0.9966
Bagging ensemble	0.7056	0.8401	–	–
Boosting Ensemble	$0.4329^*$	0.6580	–	–

$^*$Single-algorithm AdaBoost-based; less diverse than proposed method.

### Test set generalization and temporal robustness

Excellent generalization confirmed: Test MSE **0.5038 (**$R^2$** 0.9978)** (Table 5). Strict temporal validation maintains $>99\%$** explained variance** (Table 6).

**Table 5:** Test set generalization (random split).

Model	Test MSE	Test RMSE	Test $R^2$
Best GBR (tuned)	0.1770	0.4208	0.9992
Weighted Voting (Ours)	0.5038	0.7098	0.9978

**Table 6:** Temporal split validation (no leakage).

Model	Val MSE	Val $R^2$	Test MSE	Test $R^2$
Best GBR (tuned)	0.5292	0.9965	0.7711	0.9957
Weighted Voting (Ours)	1.0148	0.9932	1.0414	0.9942

**Figure 4:**
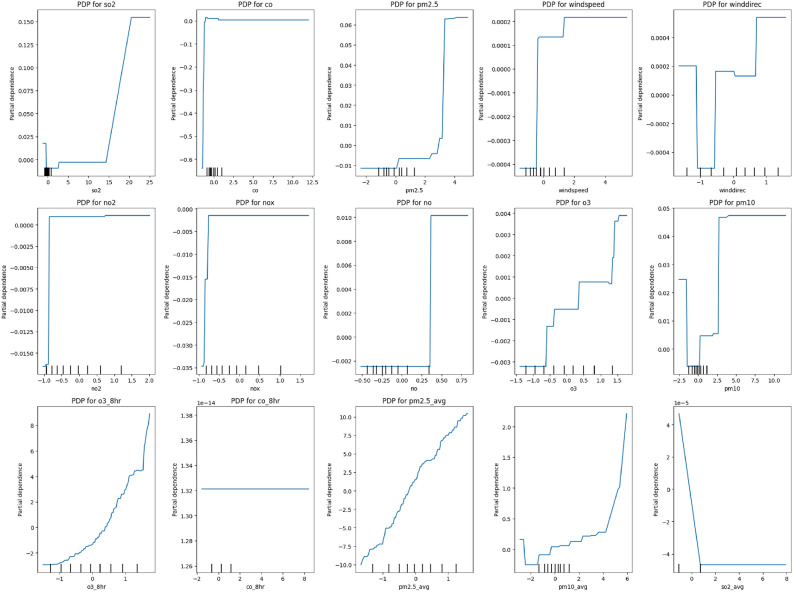
Partial dependence plots (PDPs) identify the marginal impact of the top features: $\mathrm {PM_{2.5}}$, AQI predicted by 8-hour $\mathrm {O_3}$, and $\mathrm {SO_2}$. These plots make the prediction by model more interpretable by representing and displaying non-linear interactions of features with the output.

Our weighted ensemble proves itself to be stronger than any single boosting model, with a validation MSE of 0.6553 ($\hbox {R}^2$ 0.9969) while maintaining excellent generalization (Test $\hbox {R}^2$ 0.9978) and temporal robustness ($\Delta$ $\hbox {R}^2$ = − 0.0037). This consistency in random and chronological divisions indicates that the technique can be practically deployed to operational systems to forecast AQI where the reliability of predictions in case of concept drift is critical. Such findings demonstrate the need to use ensemble models that are able to represent such complex interactions.

Figure 4 presents the Partial Dependence Plots (PDPs) to assess the marginal impact of each feature on the output of the Weighted Ensemble Regressor, thereby improving model interpretability. The findings indicate that AQI clearly benefits from features such as $PM_2.5$, $PM_{2.5}$_avg, $O_{3}$_8hr, and $PM_{10}$_avg. This observation aligns with existing research on the effects of ozone and fine particulate matter on human health. In contrast, features like *CO*, $NO_{x}$, $NO_{2}$, and wind direction show little partial dependence, implying minimal marginal contribution to the prediction when considered in isolation.

Notably, $SO_{2}$ exhibits a threshold behavior: beyond approximately 10–15 ppb (parts per billion), AQI predictions increase significantly. This nonlinearity underscores the importance of employing ensemble models capable of capturing such complex interactions. Future investigations should explore the PDP for $SO_{2}$_avg, as it reveals an unexpected inverse trend that may result from scale inversion or feature collinearity.

**Figure 5:**
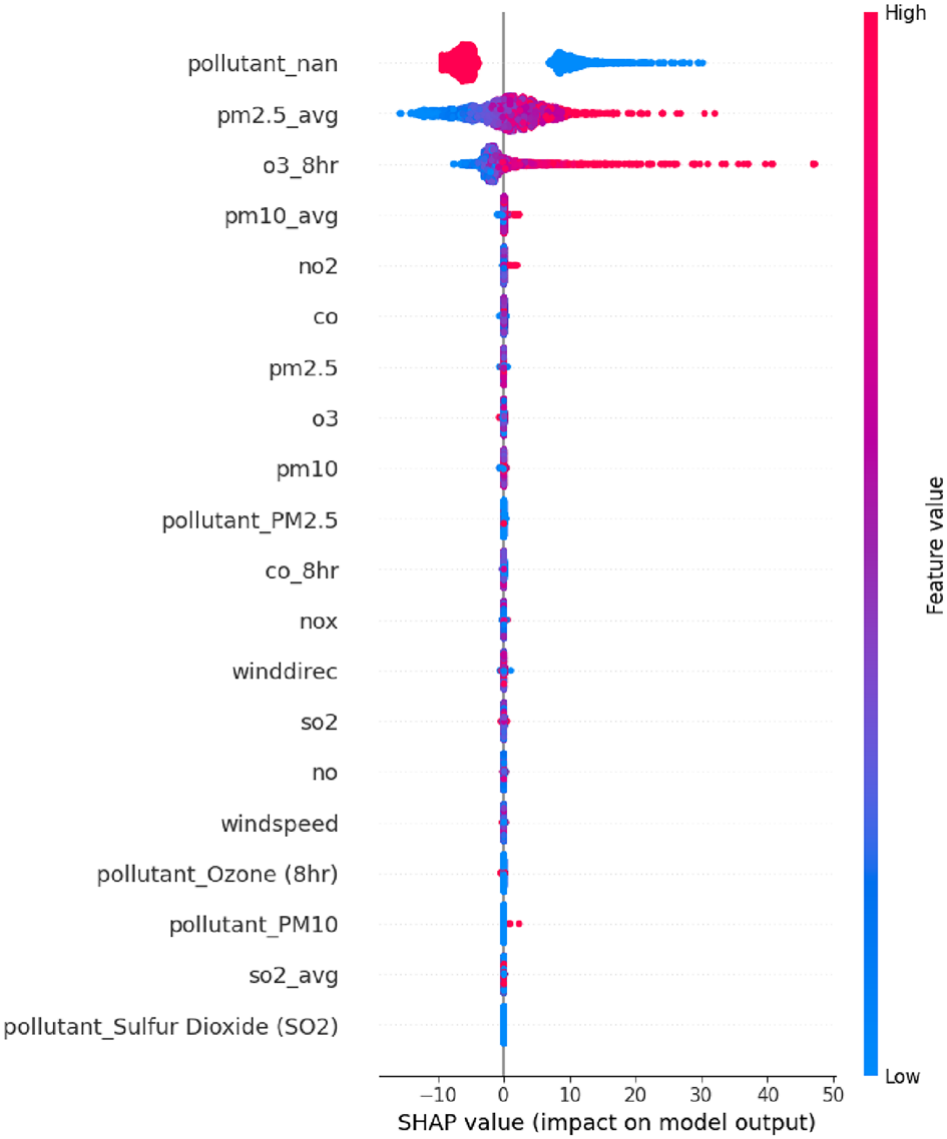
SHAP summary plot of the Weighted Ensemble Regressor, arranging input features by their influence on AQI predictions. The plot displays the magnitude as well as the direction (positive or negative) of each feature’s contribution. $PM_{2.5}$ average, $O_3$ 8-hour, and $PM_{10}$ average emerge as the most influential features, indicating the effectiveness of ensemble models in capturing complex non-linear relationships.

Figure 5 shows the SHAP summary plot of the most successful ensemble model. In line with the findings reported in the environmental science literature on particulate pollution, the average values of $\mathrm {PM_{2.5}}$, 8-hour $\mathrm {O_3}$, and $\mathrm {PM_{10}}$ were the most statistically significant in predicting the AQI. The broad spread of SHAP values across these top-performing features validates the non-linear nature of their effects and, thus, underlines the efficacy of ensemble algorithms such as Gradient Boosting and XGBoost in this research.

This study is performed on the basis of Taiwan EPA data, but the suggested ensemble framework is model-neutral and based on the universally monitored pollution and meteorological variables, which will justify the suitability in various geographic areas. The seasonal strength is partly provided by the multi-year training data that represent a range of the pollution regimes. Although the transfer between countries might bring about domain shift owing to the variation in emission origins and regulatory AQI equations, the ensemble can be fine-tuned or retrained with small local data. The concept drift, which may occur due to the change in policy or sensor advancement, can be addressed by periodically recalibrating the model and retraining on rolling-windows. It is also stated that systematic cross-domain evaluation can be one of the directions of the further work.

## Conclusion

We have outlined a weighted Voting ensemble learning models using GBR$\times$4, CatBoost$\times$3, XGBoost$\times$2, LightGBM$\times$1 to detect the air quality. This ensemble method achieved validation MSE 0.6553 (R² 0.9969) and temporal test R² 0.9942 using single-station data, outperforming 15 baselines. With a Mean Squared Error (MSE) of 0.5697, an R² score of 0.9972, and a Root Mean Squared Error (RMSE) of 0.75, the well-tuned Gradient Boosting Regressor achieved outstanding performance indicators. Advanced ensemble methods like Stacked Ensemble (MSE: 0.7070) and Weighted Ensemble (MSE: 0.6568) led to a 20–30% accuracy increased over traditional models, making them ideal for real-time AQI forecasts. Temporal validation confirmed robustness ($\Delta$R²=-0.0037) with complete-case preprocessing preserving AQI extremes. The findings demonstrated that ensemble learning models are accurate and adaptable for environmental monitoring and public health responses. Real-time integration enables early warnings, while future research could incorporate spatial graphs alongside our coordinate-agnostic tabular core for comprehensive urban deployment.

## Data Availability

The datasets used and/or analysed during the current study available from the corresponding author on reasonable request.
